# New York Clinical Reports

**Published:** 1847-07

**Authors:** B. F. Wendel

**Affiliations:** Murfreesborough, Tennessee


Art. III.—*New York Clinical Reports.* By B. F. Wendel, M.D., of Murfreesborough, Tennessee.

Dr. Wilkes's Eye Infirmary, May 7.

*Purulent ophthalmia.*—This child is about a month old, and the disease began three days after birth. This affection, occurring in the newborn child, is supposed by Mr. Lawrence to arise from some affection of the mother—leucorrhea, for instance. If the cornea is transparent, the eye is safe. The woman has been applying poultices, and these do harm, almost always producing sloughs of the cornea. The treatment is perfectly simple: Avoid poultices; give calcined magnesia for the bowels; locally, the lead wash, twenty grains to the pint, simple salve to anoint the lids at bedtime, and one grain of alum to the ounce, of which two or three drops night and morning.

*Inflammation of the membrane lining the anterior chamber.*—The iris is somewhat involved in this instance. The principal redness is just around the cornea. Solution of opium, twenty grains to the pint; cups to the temples, and pills to affect the mouth.

*Speck on the cornea.*—This child is three years old. These specks generally depend upon constitutional causes. Treatment—Ipecac. 2 grs., rhubarb 4 grs., cal. 1 gr.; sol. opii and simple salve. Let this treatment be followed by tonics.

*Amaurosis of the left eye.*—This man is about the middle age; has always enjoyed good health; has not received a blow in the face or eye, nor looked at any bright light; indeed, we can find nothing to account for the affection. What is the pathological condition in this case we cannot tell; the man is in excellent health. Our treatment hence must be somewhat empirical. Cups to the temples, and bring him under the influence of mercury.

*Congestive amaurosis.*—This man has previously suffered much pain, and he has now a vacant stare and an irregular pupil. He has been treated with alkalies, which was improper. Our plan is, free depletion, and to bring him under the influence of mercury.

*Incipient amaurosis.*—There is muddiness in the vitreous humor, and he has scintillations. Cups, and pills of calomel and jalap.

*Ophthalmia of the cornea.*—Sulphate of magnesia 1 oz., tart. emetic 2 grs., taken in divided doses; opium wash and simple salve.

*Strumous ophthalmia.*—Ipecac. and rhubarb, simple salve and the lead wash.

*Simple conjunctivitis.*—Rhubarb pill; wash, and a salve of dilute red ointment, half a drachm to the ounce.

*Pustular ophthalmia of the cornea.*—This occurs in a strumous habit, and has been an obstinate case. Iodine and hydriodate of potash or Lugol's preparation.

*Syphilitic iritis.*—Cups, calomel and opium, and infusion of opium, twenty grains to the pint.

*Morbid sensibility of the eye.*—In this case the lids do not adhere as in conjunctivitis. Pay attention to the general health; rest the organ; tonics and counter-irritation.

---

Prof. Mott's Clinic, May 8.

*Struma.*—This man has been afflicted about a year. It is a case of the first form of struma, affecting the lymphatic ganglia of the neck, and arising more from the predisposition than from anything else. He has been under the iodine treatment for some time, but with no avail. Dr. M. remarked that he had not a doubt that there was in the vegetable kingdom as certain a specific for struma as mercury is for syphilis, or quinine for miasmatic fevers. Since the iodine treatment has been found useless in this case, we will prescribe small doses of the perchloride of mercury, with the tincture of bark, and, locally, apply corrosive sublimate in the form of ointment, four grains to the ounce of lard, and cover it with oil silk.

2d. This was a case of sciatica which had existed for a number of years, and had resisted all the medicines. Issues had been lately established, and he was experiencing great relief from the remedy.

3d. *Sore mouth.*—This occurs in a woman who is nursing, but her sore mouth does not arise from this cause, for the tongue is not red and fiery, nor are there any excoriations of the membrane. Sore mouth from nursing is incurable, unless the child be weaned. Her breath shows it to be mercurial sore mouth. Since her bowels are costive, we will try a tablespoonful of sulphur in molasses morning and evening; and sage tea sweetened with honey, to which a little alum is added, for washing the mouth.

4th. *Fistula in ano.*—This is in a little boy, and has been three months in forming. Fistula in ano is a rare thing in boys, and still more rare in girls. The opening is now closed, and it will be better to let it alone. It is very seldom that the fistula heals spontaneously in the adult.

5th. A small boy who has the symptoms of stone; while urinating, the flow suddenly ceases, and he suffers much pain when the whole of the water is gone; has been frequently and carefully sounded without leading to the detection of the stone, and medicine does not relieve the symptoms. His urine abounds in crystals of lithrate of ammonia. In such cases as this, if acid abounds in the urine, calcined magnesia is the remedy; if ammoniates or earths abound, administer acids. Here we may use either nitric or muriatic acid, and will order three drops of muriatic acid three times a day in an infusion of pareira brava.

6th. An infant was brought forward which had received a burn on the forearm and hand, causing great deformity of the fingers, especially the little finger, which is turned nearly at right angles to the hand. The deformity is caused by the cicatrix, and Dr. M. thought it best to let it alone, as it occurs in a child.

7th. *Umbilical hernia.*—This little girl is quite emaciated, as if it had some hysteric trouble; she is in a state of atrophy. Its mother states that its bowels are constantly costive, for which we will prescribe the *elixir proprietatis*, a teaspoonful at night or night and morning. A truss would do no good in this case. As near as I could catch it, the professor ordered something of this kind: Take a bit of leather square or round, and spread with adhesive plaster; then take a piece of cork just fitting the aperture, cover it with buckskin, and stick it to the leather so that the convex portion of it shall go into the opening. Put on and retain.

8th. *Strumous conjunctivitis.*—Dr. M. generally succeeds in curing these things. In this case he ordered half a grain of corrosive sublimate three times a day, with the tincture of bark. He always prescribes the tincture of bark with corrosive sublimate. It is important in strumous inflammations to deplete locally while we support the system. He never saw washes of much benefit in cases of this kind, but he sometimes blisters.

9th. A German, thirty-five or forty years of age; coughs much, and spits up a great deal of mucus tinged with blood; his voice is hoarse; says he coughs most on going to bed and on rising; has night sweats, and is losing flesh daily. Dr. M. remarked that this man bore indelible marks of pulmonary disease; that we might now examine him by auscultation and percussion, but that these would not enable us to treat him any the better. Treatment: wood naphtha, elixir vitriol and paregoric, of each an ounce; of this thirty drops thrice a day in cherry-bark tea; let an issue be established where the respiratory murmur is absent, and let him have a good diet and good drink.

10th. A child four months old has a rupture. In such cases the first thing to be done is to ascertain whether the testicles are down; if they are, recommend a truss; and in this case an elastic truss is directed.

11th. *Carcinomatous ulceration of the tongue and half arches.* The man is of middle age, and states that the disease began on the side of the organ. Carcinomatous ulceration of the tongue begins on the side. The prognosis is, "*pessima est.*" If the half arches were not affected we should try extirpation, and had he been seen when this could have been done he might have been cured. The ligature is the safest method in the operation of extirpation, and causes little suffering; but if the caustic is used, it must be applied to the sound portion around the ulceration, for by applying it to the surface of a malignant disease we aggravate it.

12th. *Necrosis of the left portion of the inferior maxillary bone, caused by a blow.*—The detached portion of the bone is protruding through the gum; and I advise the man to buy a pair of forceps and pull at it every day until it gets loose and comes away.

---

PROF. PARKER'S CLINIC, May 10.

1st. *Bursa, or housemaid's knee.*—This almost always occurs in females, Dr. P. having seen it in males but few times. Its foundation is said to be in a bursa, but I do not think so, and my dissections warrant me in the belief. Bursa may be produced anywhere that there is rubbing. This man is a carpenter, and is in the habit of constantly leaning his knee against his workbench, and the swelling in him is in front of the patella and cöextensive with it. This swelling increases very slowly, and causes no inconvenience for a long time. The contained fluid varies in quality: sometimes it is albuminous, at other times there are lumps in it like boiled rice, etc. Treatment: There are different methods of treating it. It is a local difficulty, and one method is iodine or mercurial friction; another is an operation. We may incise the sack, or we may introduce a small trocar, draw off the fluid, and throw in an injection; or we may introduce a small bistoury, allow the contents to escape by the side of the knife, and then twist it about until the sack is cut in pieces; but the safest plan is to lay it freely open, and then cover with a poultice; moderate inflammation ensues, and the cavity is obliterated.

2d. *Scrofulous inflammation.*—This boy is thirteen years old, and the swelling in the leg has been in progress three months. His father sometimes suffers from rheumatism, but in other respects is a healthy man, and his mother enjoys good health. The boy has had enlargement of the lymphatic ganglia of the neck. In the leg there is swelling, redness and increase of temperature—all the local signs of inflammation; but this is not active inflammation, for it has been going on three months, and but little suppuration has yet taken place, whereas active inflammation runs into suppuration in from six to ten days. This is inflammation of the fibrous tissue, which is a common form at this age. When the periosteum is involved, so is the cellular tissue also, and this is more or less the case in the present instance. Nature would form an issue, but let us anticipate her. Apply a poultice, and as soon as suppuration has advanced, lay the swelling open. We will also give him magnesia 4 gr., rhubarb 4 or 5 gr. and blue mass ½ gr. once a day. In such cases as this, when the periosteum is involved, there is no better practice than to cut right down to the bone.

3d. *Convulsions.*—A mother brought in her child 8 months old, complaining that it had "stiffenings," and had had them as often as three or four times a week. She has had six or seven children to die about their seventh year, of the same affection. It has been in the habit of throwing up what it ate, and she has been told that the brain was affected. The child had convulsions in its first month. Dr. P. remarked that all the difficulty had arisen from the manner in which it had been fed—from some peculiarity in the mother's milk. When the brain is involved we have restlessness and squinting, which is not the case here; but we must look for the difficulty in the stomach. In a child we can learn the state of the brain at the fontanel, whether it is full or the reverse, and can feel the pulsation. If the stomach alone is at fault, cold water will be retained; but if the brain is affected, all substances are thrown up unchanged, and there is no nausea. This child has been kept at its mother's breast so long that its nervous system may have become involved. Treatment: Dentition and warm weather are coming on, and will be likely to destroy the child. It ought to be removed to the seashore, and have seabaths and friction. But little medicine is required; hydrargyrum cum creta, ipecac. and soda once in a while. Its food should be unstimulating, nutritious and digestible, and such as will not run into acetous fermentation.

4th. *Renal calculi.*—A man thirty-five years old, of sedentary habits, came in complaining of passing stones, and of much pain. He had had gonorrhea and gleet, and attributed his present difficulty to the use of lead injections in the cure of the gleet. After the cure of this affection, the gravel lay dormant for eight months; at the end of which time he took a dose of turpentine, which again excited them. Last week he passed about a quart of coagulated blood. His discharge is a ropy mucus. In disease of the kidneys the mucus floats in the water like a cloud. In prolapsus uteri, procidentia recti, etc. there is pain in the back; but if the kidneys are diseased, the stomach sympathizes. The number of stones passed indicates disease of the kidneys, but nothing else leads us to suspect them here. The pain he complains of is from the passage of the stones along the ureters; and these stones, lodging in the bladder, have given rise to a chronic cystitis, which causes the discharge. Chronic cystitis is difficult to manage, because the urine which is constantly coming into the bladder acts as an irritant; and the only way to treat it is by a retained catheter, bland tea, and nitrate of silver injections.

5th. *Hip disease?*—A man aged twenty-seven years, of scrofulous taint, complains of trouble after walking half an hour, and a pain on the inside of the knee. Pain on the inside of the knee is not indicative of hip disease. The two great symptoms of hip disease are inability to flex the limb, and pain on pressure over the trochanter; neither of which is here present. Dr. P. thinks that the limb is never longer in hip disease. The diagnosis in this case was not made out.

*Tuesday*, *May* 11.—This morning I saw Dr. Rodgers operate for a cancerous mammary gland. Previous to commencing the operation, the woman inhaled the ether between five and ten minutes without its seeming to produce the least effect, and finally she submitted to the operation without its influence. The incisions were commenced near the edge of the great pectoral muscle and made to meet at the inner circumference of the tumor close to the sternum. This woman did not seem to me to suffer any more than the man whose arm I mentioned having seen taken off at the shoulder, and who was under the influence of the sedative.

---

Prof. Parker's Clinic, May 17.

1st. *Lumbar or psoas abscess.*—This girl is eight years old. Three years since she fell from some height upon her back, which caused her much pain and difficulty in walking for some time; but she gradually recovered from it. She is now in pretty good health, though at times peevish. A month or more after the accident she had measles, and began to be lame a year after suffering with that disease. She has not much scrofulous tendency naturally, and had it not been for the fall, would probably have escaped it. There was some enlargement of the lymphatic ganglia of the neck after the fall. She is of the right age to suffer from this form of scrofula. Scrofulous disease attacks first the skin and lymphatic ganglia, secondarily the bones, especially their spongy portion, and lastly the internal organs. There is, however, an exception to this last; tubercles of the brain occur at an early period of life. Had she fallen upon the trochanter instead of the back, she would probably have had hip disease. When one has a scrofulous predisposition and on this a constitutional affection, the tubercular deposition will be awakened; for example, if one is predisposed to a deposit of tubercles in the brain and has scarlet fever, they will be developed. Pain in the limbs is one of the first symptoms; but the mother says that this child has not had any. The lumbar vertebræ are involved; the body of one of them is absorbed, and one or two more are affected. Suppuration has taken place, and the matter has passed under the psoas muscle, under Poupart's ligament, and pointed on the inside of the thigh. Lumbar abscess may occur about the psoas muscle without involving the bone; or this may be primarily involved; or the disease may work up and involve the bone. In this case it has commenced in the bone and worked its way down. In this instance the symptoms and diagnosis are clear; but they are not always so. A person may come to you complaining of pain and uneasiness on the inside of the knee, and a sense of weight in the loins: in hip disease the pain is on the outside of the knee, and passing your hand over the abdomen you may discover a tumor; in hernia the vessel is on the inside, but in abscess generally on the outside; coughing gives an impulse to both.—Prognosis unfavorable.

*Treatment.*—Shall it be allowed to remain as it is, and let nature discharge it, or shall we open it? Leave it to nature. Constitutional treatment is wholly embodied in this: We must sustain the child; medication is worth little. Let her take iodide of iron, and have good air, good diet, etc. Locally neither blisters nor ointments would do any good, though, were the tumor not so large and the skin not involved, a blister might be beneficial. The old practitioners plunged their lancets into this kind of tumor and discharged its contents, and found that the patient finally sunk; they then went to the opposite extreme. Mr. Abernethy thought that we might effect a cure if we could contrive to draw off a portion of the fluid at a time, by making an opening at the edge of the swelling, closing it, and then applying moderate pressure, and pursuing this course for some time. And Prof. Parker thought this plan might succeed.

2d. *Circocele.*—This is in a young man, and is of three months' standing. The enlargement is on the left side at the upper and back part of the testicle, which is almost always the case. Circocele is worse in the after part of the day, and on warm than on cold days. It may be confunded with hernia. We can feel the neck of a hernial tumor, while the other feels like worms. Let the patient lie down, and if the hernia is reducible, and we press upon the abdominal ring, the hernia will not return on rising; but if it be circocele, it will rather be increased in size. Treatment: The palliative plan is generally sufficient. Bring the tumor against the external ring, apply the bag truss, and give the cold dash, which will cause the vessels to contract and give tone to them. It is important also that the bowels be kept free. There are many operations for circocele—the caustic; the thread; hot needles; the forceps, seizing the skin and vein with them, and retaining; cutting away a portion of the scrotum; exsection; tying the veins; tying the arteries, etc. Dr. Parker's plan is to seize the skin and vein between two fingers, pass a needle under it and the twisted suture around it, which he lets remain in from one to four days. An operation is to be avoided if possible, and Dr. P. would not operate in a bad constitution. The danger of tying veins is not by any means so great as some represent. As for the causes of circocele, it is difficult to point to them; onanism and excessive venery are said to cause it; Dr. P. had a case in which it was produced by wearing very tight pantaloons.

3d. *Halt.*—This man, "to save himself," jumped, and the lower portion of the patella separated from the upper. This accident occurred some two years ago, and at the time he was not able to rise or stand up. The portions of the patella and their uniting ligament can be felt; they are an inch and a half or two inches apart. He feels pain after walking but little, and in stepping is obliged to throw the foot of that leg upwards. Not much can be done in such cases as this: the cold dash, and any apparatus that will keep the portions of the patella near each other.

4th. *Cutaneous cancer.*—A woman aged thirty years, of scrofulous family, presented herself with an open sore near the external angle of the eye; it had been progressing for seven or eight years, and "commenced as a wart." She had done nothing for it save applying a common plaster. This is an original disease of the skin, and the question as to what it is will lie between *lupus* and *cancer:* it has a hard, scirrhous feel, and we diagnosticate it to be *cutaneous cancer*. The lymphatic ganglia situated in front of the lobe of the ear were enlarged; Dr. P. remarked that in enlargement of the lymphatic ganglia from cancerous disease they were not tender, but that in the same enlargement from a scrofulous taint they were quite tender. We might excise this and bring up a portion of skin to cover the wound.

5th. *Lateral curvature of the spine.*—A chambermaid, æt. 18 years, who has been in the habit of carrying buckets of water, and who has never had her "periods," has left lateral curvature; the curvature is generally to the right. She was obliged to give up her occupation on account of a pain in the left shoulder and heart, which, however, ceases when she lies down. The curvature seems not to be in proportion to the displacement of the shoulder. Dr. P. has no confidence in the squeezing treatment. It generally occurs in persons who have not exercised sufficiently, and the plan is to let her have plenty of exercise, laboring mostly with the arm that is not pushed up. Pulling up by ropes is an excellent plan; add to this proper diet, etc. Iron has been recommended in these cases; but the long and short of it is, there *will* be more or less deformity. Dividing the muscles has been recommended and practiced, but I have not much faith in it. It is desirable to bring about the menses. As for her pain, that is nervous, and some liniment or tincture of iodine may be applied for it.

6th. *Scrofulous enlargement of the ankle joint.*—A child, aged two years, whose father a short time before died of consumption, has a scrofulous enlargement of the ankle joint. In this case there is chronic synovitis of a scrofulous nature. The treatment must be of a constitutional kind—a tonic course, the country, good diet, and plenty of exercise. We may give the iodide of iron internally, and, locally, apply the tincture of iodine, covering the part with oil silk. In these cases we generally find an enlarged abdomen and thick upper lip, etc.

7th. A fine, healthy looking girl, about 12 years old, whose bowels are regular, but whose appetite is variable, has epileptic fits. She had suffered an attack of scarlet fever, which the mother supposed was the cause of the first convulsions; she has also passed worms, but the fits came on three or four months after this occurrence without any assignable cause. During a fit she froths at the mouth, her eyes roll, and her hands are clenched. Where the difficulty is, in this case, it is difficult to tell. These convulsions arise from irritation in the abdomen oftener than from anything else, and as her tongue is deeply coated, we will direct our efforts in that way. Let her take an aloetic and blue pill every night, and live on a vegetable diet. Her hair must be cut short, and the cold dash applied to the head and down the spine, and, as her feet are cold, to them also.

---

PROF. MOTT'S CLINIC, May 22.

1st. A boy presented himself who had now sores on his face and scabs on the back; though he had often had them on his head, he had none now, and they were at times more or less all over the body. These had been coming and going for eight years. Prof. M. thinks it is not eczema, since it is of so long standing and is all over the body; and he remarked that we could cure it much more easily than name it. Treatment: Muriate of mercury two grains to an ordinary sized bottle of sarsaparilla, of which take a teaspoonful night and morning, and an ointment to rub the affected part with.

2d. *Caries of the spine.*—A boy who had his back hurt six months ago, now has caries of the spine, of which we see plenty of cases here. This is a tuberculous disease of the vertebræ, and an affection that is not well understood. It is frequently treated by pressure, and this violent pressure leads to ulceration. This is a totally different disease from the one requiring mechanical treatment at first, for this must be arrested before the mechanical treatment is applicable. Treatment: Counter-irritation, either by setons or issues, it is immaterial which; and the muriated tincture of iron internally. This boy has weakness of the limbs, and in these cases we often have cramps in the legs and twitching of the muscles, from compression of the spinal marrow. When the cure is effected, there will be anchylosis, of course, and no machine must be applied until this takes place.

3d. A young man came in complaining of his ankle joint, which is not swollen at all, and of weakness at the knee, and pain after walking. The difficulty was occasioned by stepping on a round stone four months ago, which rolled and twisted his ankle. There are many of these pains the nature of which it is difficult to ascertain, and Dr. M.'s plan is to keep the part from sight as much as possible; therefore we will cover it with emplastrum hydrargyri cum gummo ammoniaco.

4th. This is a woman with an unhealthy ulcer on the leg, of twelve years' standing, and for which she has tried many things. Dr. M. thinks it as well to call all ulcers either healthy or unhealthy, and that the varieties of these are of little consequence. The solution of this is that there is vitiation, and there is vitiation because the granulations are swept away by the inflammation as fast as formed. Our treatment must be both local and constitutional, to change the character of the sore. Yeast and milk, or yeast and flaxseed poultice to reduce the inflammation, and a slight stimulus might be beneficial; a tonic course, and as soon as the ulcer is nearly healed establish an issue. It is absolutely necessary that this woman give up exercise.

5th. This is a case which the professor looked at before coming in, and considers it as *sui generis.* The little boy is six or seven years old, and had fits every month or two for two years; at the beginning of this spring he began to have them more frequently, and now has four or five a week; he froths at the mouth during a fit, screams and moans hard after it, and has sudden twitchings of the muscles through the day; he cannot balance himself in walking. This is an affection of the cerebro-spinal axis, and much of it is connected with the cerebral mass; but whether there is inflammation of the arachnoid and effusion or *rammollissement*, I am unable to say. The treatment must be somewhat empirical. The cases which are cured of epilepsy are sympathetic. There is an issue already established in the back of the neck, and we will order two drops of Fowler's solution three times a day.

6th. A girl who has not had her courses for a long time, and whose general health is not good, complains of dizziness, and has fits two or three times a day, in which she is perfectly stiff and insensible. She has had them about two years, and says they are worse about the time of her periods. This is a case of symptomatic catalepsy, connected with obstructed catamenia, and calls for the use of emmenagogues, if there be any such medicines. Her general system must be strengthened—an aloetic and ferruginous pill, air and exercise. The French plan of bleeding in the foot might be useful, or a few leeches within the labia majora.

I have had the good fortune to receive an appointment in the Bellevue Hospital, and my communications in future will relate chiefly to cases observed in that institution. At present the hospital is crowded to great excess by the paupers arriving in every ship from Ireland, and ship fever is prevailing to a fearful extent. Evils, it is said, always go in crowds, and it is emphatically true of famine, which brings in its train a host of diseases. From the public prints you will learn that great numbers are arriving here every week from the old countries in a state of starvation, and the hospital reports show that many of them only come to find a grave.

New York, June 1, 1847.

